# Characterization, Classification and Authentication of Turmeric and Curry Samples by Targeted LC-HRMS Polyphenolic and Curcuminoid Profiling and Chemometrics

**DOI:** 10.3390/molecules25122942

**Published:** 2020-06-26

**Authors:** Nerea Núñez, Oscar Vidal-Casanella, Sonia Sentellas, Javier Saurina, Oscar Núñez

**Affiliations:** 1 Department of Chemical Engineering and Analytical Chemistry, University of Barcelona, Martí i Franquès 1-11, E08028 Barcelona, Spain; oscarvidalcasanella@gmail.com (O.V.-C.); ssentellas@hotmail.com (S.S.); xavi.saurina@ub.edu (J.S.); 2 Research Institute in Food Nutrition and Food Safety, University of Barcelona, Recinte Torribera, Av. Prat de la Riba 171, Edifici de Recerca (Gaudí), Santa Coloma de Gramenet, E08921 Barcelona, Spain; 3 Serra Húnter Fellow, Generalitat de Catalunya, E08007 Barcelona, Spain

**Keywords:** targeted LC-HRMS analysis, turmeric characterization, polyphenols, curcuminoids, principal component analysis, partial least squares-discriminant analysis

## Abstract

The importance of monitoring bioactive substances as food features to address sample classification and authentication is increasing. In this work, targeted liquid chromatography coupled to high-resolution mass spectrometry (LC-HRMS) polyphenolic and curcuminoid profiles were evaluated as chemical descriptors to deal with the characterization and classification of turmeric and curry samples. The profiles corresponding to bioactive substances were obtained by TraceFinder^TM^ software using accurate mass databases with 53 and 24 polyphenolic and curcuminoid related compounds, respectively. For that purpose, 21 turmeric and 9 curry samples commercially available were analyzed in triplicate by a simple liquid–solid extraction procedure using dimethyl sulfoxide as extracting solvent. The obtained results demonstrate that the proposed profiles were excellent chemical descriptors for sample characterization and classification by principal component analysis (PCA) and partial least squares-discriminant analysis (PLS-DA), achieving 100% classification rates. Curcuminoids and some specific phenolic acids such as *trans*-cinnamic, ferulic and sinapic acids, helped on the discrimination of turmeric samples; polyphenols, in general, were responsible for the curry sample distinction. Besides, the combination of both polyphenolic and curcuminoid profiles was necessary for the simultaneous characterization and classification of turmeric and curry samples. Discrimination among turmeric species such as *Curcuma longa* vs. *Curcuma zedoaria*, as well as among different *Curcuma longa* varieties (Alleppey, Madras and Erode) was also accomplished.

## 1. Introduction

Currently, society’s interest in consuming natural food products rich in bioactive phytochemicals with healthy properties is increasing, giving place to the production of nutritional supplements and functional foods based on those bioactive substances. Turmeric (*Curcuma longa*), which is a plant-derived spice related to the ginger family (Zingiberaceae), is among the natural food products that have been widely recognized for their medicinal properties [1,2,3,4,5]. Consequently, turmeric is one of the most popular traditional medicinal herbs with a wide range of pharmacological activities such as antioxidant, antimicrobial, antimalarial, anti-inflammatory, anti-tumoral and anti-aging properties [6,7]. These beneficial health properties are mainly attributed to the presence of curcuminoids, being curcumin (diferuloylmethane) the most important one [6,8,9,10]. In addition to curcuminoids, other healthy substances such as phenolic acids, flavonoids, terpenoids, phenylpropanoids and sesquiterpenes are present in turmeric products [10,11]. Besides, turmeric is also a highly appreciated spice used in the preparation of curries, especially in India and other Asian countries, because of its flavor and color, providing a yellow hue to the dishes [12,13,14].

Several analytical methodologies have been proposed for the characterization, identification and determination of curcuminoids in turmeric samples [3,8,15]. Some of them are based on spectral data from ultraviolet (UV) [16,17], fluorescence [18], Fourier transform infrared (FTIR) [16,19,20] or nuclear magnetic resonance (NMR) [16,21,22,23,24] spectroscopies. Electrochemical characterizations and determinations of curcuminoids in turmeric samples by bare and modified electrodes [25], such as graphene-modified [26] and multiwalled carbon nanotube modified [27] electrodes, have also been reported. Anyway, separation techniques are the most common choice for the characterization and determination of bioactive substances, including curcuminoids. For example, capillary electrophoresis (CE) has been applied to the determination of curcuminoids and related compounds in turmeric products based on different CE modes such as capillary zone electrophoresis, non-aqueous capillary electrophoresis or micellar electrokinetic chromatography [28,29,30,31]. Gas chromatography coupled to mass spectrometry (GC–MS) has also been used for the characterization of powdered turmeric [32] and turmeric oil [33] samples. Nevertheless, liquid chromatography (LC) with UV detection (LC-UV) [34,35,36,37,38,39,40] and coupled to mass spectrometry (LC–MS) [32,41] are currently the techniques of choice. Besides, the great chemical diversity among bioactive substances, including polyphenolic and curcuminoid compounds, and the wide range of concentrations in which these phytochemicals can be found in turmeric and related products make liquid chromatography coupled to high-resolution mass spectrometry (LC-HRMS) the most appropriate strategy for characterization, identification and authentication purposes [42]. This is mainly due to the advantages associated with the high-resolution and accurate mass measurements attainable with these instruments. Time-of-flight (TOF) and Orbitrap mass analyzers, but especially their hybrid configurations, such as quadrupole-time-of-flight (Q-TOF), quadrupole-Orbitrap (Q-Orbitrap) and linear trap quadrupole-Orbitrap (LTQ-Orbitrap), because of their fragmentation capabilities, are the most frequently HRMS instruments used for the identification of polyphenols and curcuminoids in turmeric products [43,44,45,46,47,48]. In addition, ambient mass spectrometry techniques, such as direct analysis in real time-mass spectrometry (DART-MS), have also been described for the spatial localization of curcumin and the rapid screening of the chemical composition of turmeric rhizomes [49].

The characterization and authentication of a wide range of food products are often carried out in a straightforward way using instrumental techniques such as FTIR spectroscopy and HPLC with UV detection, which are especially suitable for the analysis of large sets of samples. Compared to these techniques, LC-HRMS is undoubtedly a more complex and expensive choice. Regardless, LC-HRMS platforms are increasingly used in food analysis because of their excellent performance to find out specific markers of interest for sample discrimination and authentication. Thus, the research based on exact mass data can be exploited to detect, almost unambiguously, the occurrence of a given molecule as well as to disregard many others. As a result, more robust evidence can be obtained from those significant descriptors that contribute to discrimination between samples and classes. The massive amount of data generated from the spectroscopic and chromatographic techniques, such as in the case of LC-HRMS, may hinder the extraction of overall conclusions on characterization and authentication issues. Under these circumstances, the use of chemometric methods is almost essential to recover the underlying information from the data. In the case of turmeric products, their classification and authentication have also been tackled in various works. For that purpose, chemometrics is used for the assessment of patterns and relationships among samples, as well as to establish discriminant sample chemical descriptors, using both targeted and non-targeted approaches. Non-targeted fingerprinting approaches rely on the analysis of complex instrumental responses without assuming any previous knowledge of relevant or irrelevant sample components [50]. For example, Di Anibal et al. [51] determined the adulteration of several spices, including turmeric and curry, with Sudan I-II-III-IV dyes by UV–visible spectroscopy and multivariate classification techniques such as partial least squares regression-discriminant analysis (PLS-DA), among others. Windarsih et al. employed NMR spectroscopy fingerprinting in combination with principal component analysis (PCA), PLS-DA and orthogonal projections to latent structures-DA (OPLS-DA) for the authentication of *Curcuma longa* adulterated with other cheaper curcuma species such as *Curcuma manga* or *Curcuma heyneana.*

Targeted approaches rely on the determination of known selected compounds, often belonging to the same family or with a similar structural feature. The concentrations of these targeted compounds can be used as tentative food markers to address food authenticity. This approach implies the quantitative analysis using standards for each targeted component. Nevertheless, this can be a challenging task because of the sample matrix complexity when dealing with food products, especially due to the presence of unknown interfering compounds [50]. Apart from quantitative methods, instrumental signals such as chromatographic peak areas of known selected compounds are a good alternative for food characterization and classification, also providing chemical information related to bioactive substances [22,23,24]. In this regard, polyphenolic and curcuminoid profiling by HPLC-UV has been reported for the characterization of turmeric and curry samples [39,40]. Finally, the use of DNA barcoding by polymerase chain reaction (PCR) amplification was also proposed for the detection of plant-based adulterants (cassava starch, wheat, barley and rye) in turmeric powder samples [52].

In the present work, targeted LC-HRMS using a Q-Orbitrap mass analyzer was proposed for polyphenolic and curcuminoid profiling. The applicability of the obtained LC-HRMS polyphenolic and curcuminoid profiles as sample chemical descriptors to classify and authenticate turmeric and curry samples was evaluated. With this aim, a total of 30 commercially available turmeric and curry samples were analyzed with the proposed methodology after a simple sample extraction procedure. The approach relied on the study of signal intensities of peaks associated to the target compounds so that the time-consuming step of analyte quantification was not necessary; even data from unknown species (e.g., without available standards or molecular structures not fully elucidated) can be considered. In more detail, the whole method is composed of several steps, as follows: (i) Sample treatment was based on solvent extraction with DMSO, specially chosen to provide the richest compositional profiles dealing with curcuminoids and polyphenols. (ii) LC-HRMS analysis of extracts in which separation conditions were established to maximize the number of peaks in the chromatograms while HRMS conditions were focused on curcuminoid and polyphenol detection. (iii) Sample polyphenolic and curcuminoid profiling was carried out by screening the analyzed samples using the TraceFinder^TM^ software (ThermoFisher Scientific) with home-made accurate mass databases including 53 polyphenolic compounds previously characterized with commercially available standards [53] and 24 curcuminoid related signals from commercially available standards or data reported in the literature. (iv) The obtained peak signal profiles were then used as the source of analytical information for PCA and PLS-DA. Ion intensities were arranged in a data matrix, with rows representing the samples and columns the target analytes. The resulting plots of scores and loadings were interpreted to extract the underlying chemical information concerning sample patterns and relevant descriptors. Classification models were established as well from which unknown turmeric and curry samples were assigned to their corresponding classes.

## 2. Results and Discussion

### 2.1. Targeted LC-HRMS Polyphenolic and Curcuminoid Profiling

This work aims to evaluate the suitability of targeted LC-HRMS polyphenolic and curcuminoid profiles as sample chemical descriptors to address the characterization and classification of turmeric versus curry samples. For that purpose, a simple liquid–solid extraction procedure to recover polyphenolic and curcuminoid compounds with dimethyl sulfoxide (DMSO) as the extracting solvent was proposed [40].

**Figure 1:**
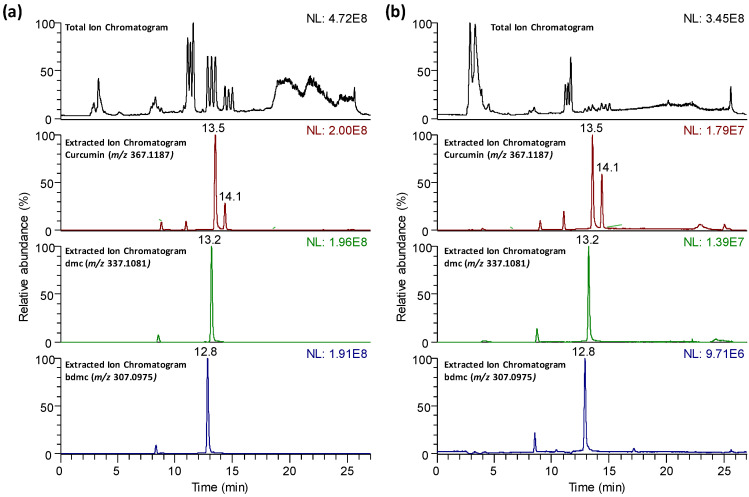
Reversed-phase liquid chromatography coupled to high-resolution mass spectrometry (LC-HRMS) total ion chromatograms and extracted ion chromatograms of curcumin (*m/z* 367.1187, retention time, RT, 13.5 min and RT 14.1 min—tautomeric forms), dmc (*m/z* 337.1081, RT 13.2 min) and bdmc (*m/z* 307.0975, RT 12.8 min) for (**a**) a turmeric (Biospirit brand) sample and (**b**) a curry (Hacendado brand) sample.

It is well known that polyphenolic and phenolic acid substances are typically analyzed by reversed-phase chromatography, using water and methanol or acetonitrile (acidified with 0.1% formic acid) as the mobile phase components [42]. Similar approaches have also been described for the chromatographic determination of curcumin and related compounds [8,15,39,40]. Therefore, turmeric and curry LC-HRMS polyphenolic and curcuminoid profiles were obtained by C18 reversed-phase column and using a gradient elution program with 0.1% formic acid aqueous solution and acetonitrile with 0.1% formic acid as the mobile phase components. Figure 1 depicts, as an example, the LC-HRMS total ion chromatogram (TIC) and the extracted ion chromatograms of curcumin (*m/z* 367.1187, retention time, RT, 13.5 min), demethoxycurcumin (dmc, *m/z* 337.1081, RT 13.2 min), and bisdemethoxycurcumin (bdmc, *m/z* 307.0975, RT 12.8 min), for (a) a turmeric sample and (b) a curry sample. As can be seen, important differences in the TIC profile and signal intensities are observed. As expected, curcuminoids were more abundant in turmeric samples than in curry ones.

Targeted LC-HRMS polyphenolic and curcuminoid profiles were then obtained by processing the raw chromatographic data with TraceFinder^TM^ software using two accurate mass databases combining 53 polyphenolic and 24 curcuminoid compounds (the confirmation criteria employed are described in Section 3.4—data analysis). Only peak areas of compounds detected with a signal higher than an established threshold (1.0 × 10^5^) and accomplishing all the confirmation criteria defined in the screening software were considered as positive matches to be included in the polyphenolic and curcuminoid profiles. As an example, Table 1 summarizes the TraceFinder^TM^ profiling report obtained for a turmeric (Biospirit brand) sample. In this specific sample, 17 polyphenols and 14 curcuminoids were detected and confirmed. As can be seen, curcuminoid peak areas in turmeric samples are meaningfully higher than those of polyphenolic ones, being curcumin, dmc and bdmc the most intense peaks with signals higher than 1.4 × 10^9^.

### 2.2. PCA

The targeted LC-HRMS polyphenolic and curcuminoid profiles of all the analyzed samples and quality controls (QCs) were employed as the source of potential sample chemical descriptors for exploratory study by PCA. A data matrix with a dimension of 101 (sample extracts + QCs) × 135 (peak areas of the *m/z* values at the different retention times detected) was obtained. Data was autoscaled to provide similar weights to all the variables. Figure S1 shows the PCA scores plot of principal component 1 (PC1) vs. PC2 depicting the distribution of samples and QCs. As can be seen, QCs are not correctly grouped, showing a clear dependence on the injection order within the sequence, from QC1 (analyzed at the beginning of the sequence), located at the top-right area of the plot, to QC11 (analyzed at the end of the sequence), located close to the center area of the plot. This quite poor reproducibility could be related to changes in the electrospray ionization (ESI) efficiency with time, caused by the complexity of the sample matrices analyzed. Consequently, the sample distribution map should be affected by the same problem, so that corrective mechanisms should be implemented to minimize the impact of the decrease in sensitivity throughout the analysis series. This correction will rely on QC injection performances.

**Figure 2:**
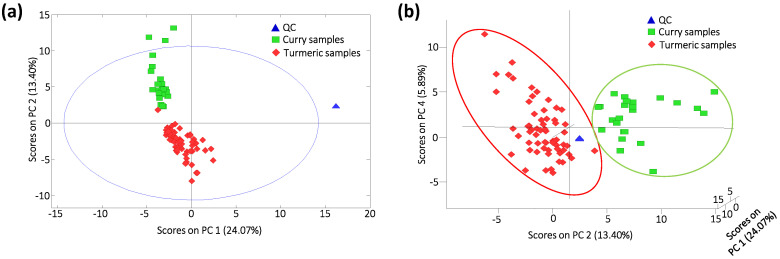
Principal component analysis (PCA) score plots of (**a**) principal component 1 (PC1) vs. PC2 and (**b**) PC1 vs. PC2 vs. PC4 when using corrected targeted LC-HRMS polyphenolic and curcuminoid profiles as sample chemical descriptors. A total of 4 PCs were used to build the model.

For that purpose, polyphenolic and curcuminoid peak areas were divided by those obtained in the closest QC (whereas each QC peak area was divided by itself). With this QC normalization, all QCs will appear exactly in the same plot position (all their profile variables are 1), while the loss of signal with time in the analyzed turmeric and curry samples caused by the changes in the ESI ionization efficiency is corrected. As a result, Figure 2a shows the PCA score plots of PC1 vs. PC2 for all the samples using the corrected profiles. As can be seen, very acceptable discrimination among the analyzed samples was obtained, being PC2 the main responsible for the sample distribution, with curry samples being located at the top of the plot, and turmeric samples at the bottom of the plot, except for one sample that seems to be overlapped with the curry samples. However, when considering an additional PC, clear discrimination between both groups of samples is achieved, as can be observed in Figure 2b showing the 3D PCA score plot of PC1 vs. PC2 vs. PC4.

### 2.3. Supervised Sample PLS-DA Classification

**Figure 3:**
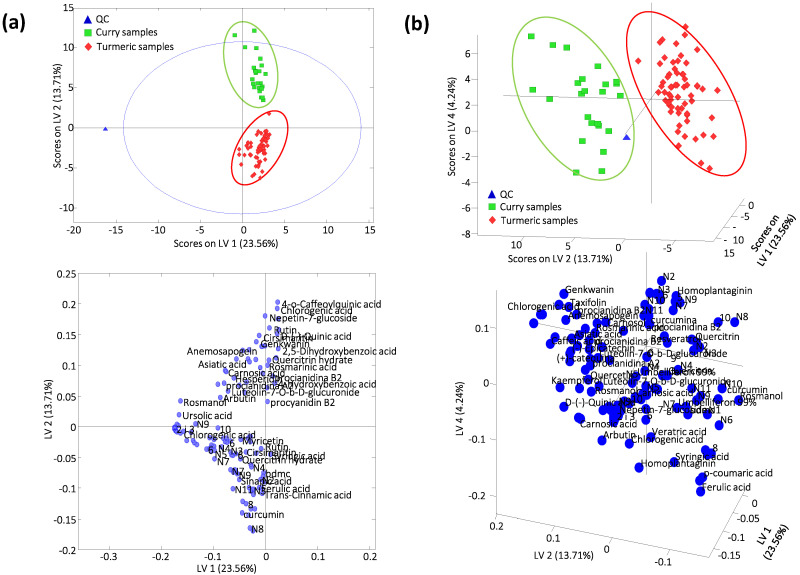
Partial least square regression-discriminant analysis (PLS-DA) score (above) and loading (below) plots of (**a**) latent variable 1 (LV1) vs. LV2 and (**b**) LV1 vs. LV2 vs. LV3 when using corrected targeted LC-HRMS polyphenolic and curcuminoid profiles as sample chemical descriptors. A total of 4 LVs were used to build the model. Enlargements of loadings plots including full description names is provided in Figure S2 (Supplementary Materials).

LC-HRMS polyphenolic and curcuminoid profiles as sample chemical descriptors were also evaluated by employing a supervised classificatory chemometric method such as PLS-DA. In this case, the corrected X-data matrix, standardized by the QC intensities, was used (i.e., one previously employed in the PCA study), while the Y-data matrix included the membership (turmeric vs. curry) of each analyzed sample. In this case, a total of 4 latent variables (LVs), obtained by cross-validation (CV) error from a Venetian blind approach, were required to establish the PLS-DA model. Figure 3 shows the PLS-DA score (sample distribution) and loading (variable distribution) plots of (a) LV1 vs. LV2 and (b) LV1 vs. LV2 vs. LV4 when the corrected polyphenolic and curcuminoid profiles were used as sample chemical descriptors for sample classification. Perfect discrimination between both groups of samples was achieved, being LV2 the main responsible for this differentiation. Curry samples show positive LV2 values, while turmeric samples display negative LV2 values.

The study of the PLS-DA loading plots (Figure 3) revealed the most discriminant targeted bioactive compounds for each group of samples. Enlargement of the loading plots, with full name descriptions, are shown in Figure S2 (Supplementary Material). As can be seen in Figure S2, 166 variables were employed to establish the chemometric models. It should be noted that the same *m/z* values were detected at several retention times. This was due to the presence in the sample of both aglycones and some derivatives (i.e., glycosylated derivatives and other adducts, in the case of polyphenolic compounds). These related compounds were chromatographically separated. However, they could suffer in-source fragmentation in the ESI source yielding the ion (*m/z* value) corresponding to the aglycone and, consequently, TraceFinder™ provides a match at a different retention time. Besides, most of the curcuminoids adopt two forms due to keto-enol tautomerism. The enol and keto forms have the same *m/z* value but can usually be separated chromatographically. This is a well-known phenomenon and has been documented within several curcuminoids, especially curcumin [54,55]. In fact, we detected curcumin in many of our samples as two peaks having retention times of 13.5 and 14.1 min, which likely correspond to the enol and keto forms. It is difficult to designate each peak as being the enol or keto form of curcumin. Such differentiation is not relevant to, and is beyond the scope of this study.

Curcuminoid compounds in general, and mainly curcumin, dmc and bdmc, and other derivatives such 8, N2, N3, N7, N8, N9 and N11, are the main responsible for the discrimination of turmeric samples. In contrast, polyphenolic compounds differentiates the curry samples. Among them, 4-O-caffeoylquinic acid, chlorogenic acid, nepetin-7-glucoside, rutin and D-(-)-quinic acid seem to be relevant markers. However, some polyphenolic compounds such as *trans*-cinnamic, ferulic and sinapic acids also help on the discrimination of turmeric samples. Even though curcuminoids are found at higher intensities in turmeric samples than polyphenols, discrimination of both turmeric and curry samples was only accomplished when both polyphenolic and curcuminoid profiles were used (see Figure S3 depicting the corresponding PCA score plot when using only curcuminoid profiles).

**Figure 4:**
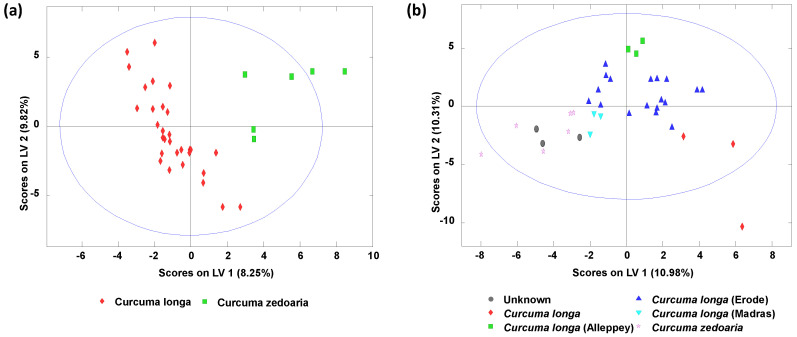
Partial least square regression-discriminant analysis (PLS-DA) score plots of LV1 vs. LV2 when using corrected targeted LC-HRMS polyphenolic and curcuminoid profiles as sample chemical descriptors for (**a**) classification of *Curcuma longa* vs. *Curcuma zedoaria* turmeric samples, and (**b**) when also considering all the different *Curcuma longa* varieties. A total of 4 LVs were used to build the model.

PLS-DA was also applied to try to discriminate among turmeric varieties based on LC-HRMS polyphenolic and curcuminoid profiles. Figure 4 shows the obtained PLS-DA score plots of LV1 vs. LV2 when comparing (a) *Curcuma longa* vs. *Curcuma zedoaria* species and (b) when also considering the different varieties of *Curcuma longa*. Despite the reduced number of samples available, good discrimination among them was observed. For example, when studying the PLS-DA classification of *Curcuma longa* vs. *Curcuma zedoaria* (Figure 4a), two groups were distinguished, being LV1 responsible for the sample discrimination. Samples belonging to the *Curcuma zedoaria* exhibit positive LV1 values, being located at the right of the plot, while *Curcuma longa* samples are located at the left of the plot. Besides, when considering all the *Curcuma longa* varieties (Figure 4b), very acceptable discrimination among the analyzed turmeric samples was also observed. Again, LV1 is responsible for the discrimination among *Curcuma longa* and *Curcuma zedoaria* samples, although when considering all these varieties, *Curcuma zedoaria* samples display negative LV1 values and *Curcuma longa* samples positive ones. Again, and despite the low number of samples available for some varieties, separation among the different *Curcuma longa* samples (Alleppey, Madras and Erode) was also observed. In this case, both LV1 and LV2 were involved in the achieved discrimination. For example, Alleppey samples were located at the top-center area of the PLS-DA plot, with LV2 as positive values, and then as LV2 values decreased, Erode and Madras samples could be found. The sample represented with a rhombus showed this peculiar behavior, far away from other similar samples, since it was aged beyond the expiration date. Finally, the replicates of a turmeric sample of an unknown variety (NAAI commercial brand) are overlapped with samples corresponding to *Curcuma zedoaria*, although a high number of samples will be necessary to confirm its authenticity.

These results demonstrate that the proposed 53 phenolic and polyphenolic acids and 24 curcuminoid related compounds seem to be appropriate to address the characterization and classification of turmeric and curry samples, as well as among the different turmeric varieties.

### 2.4. PLS-DA Method Validation

**Figure 5:**
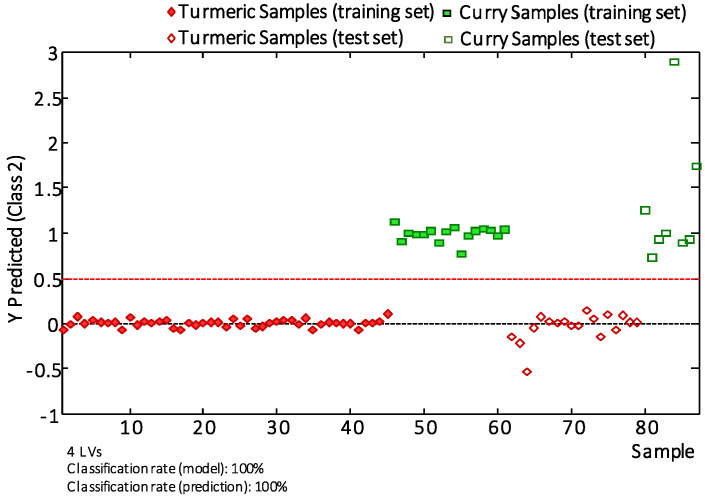
Y predicted 1 vs. samples scores plot for turmeric vs. curry samples. Filled and empty symbols correspond to calibration and validation sets, respectively. The number of LVs employed to generate each classificatory model and sample classification rate are also indicated.

The applicability of the proposed methodology to classify turmeric and curry samples by PLS-DA was evaluated. With this aim, the PLS-DA model studied was established by using 70% of the samples randomly selected for each group as the calibration set, and the remaining 30% of the samples were then employed as the “unknown” set for validation purposes. Figure 5 shows the classification plot obtained using 4 LVs to build the model.

As can be seen, a 100% classification rate was obtained for both calibration and prediction models, showing the great feasibility of LC-HRMS polyphenolic and curcuminoid profiles as sample descriptors for the characterization, classification and authentication of turmeric and curry samples.

## 3. Materials and Methods

### 3.1. Chemicals and Standard Solutions

All the reagents, chemicals and standards used in this research were of analytical grade. The 53 phenolic compounds including phenolic acids, benzoic acids, phenolic aldehydes, cinnamic acids, phenolic terpenes, flavones, flavanols, proanthocyanidins and stilbenes were purchased from Sigma-Aldrich (Steinheim, Germany). Curcumin (cur, 98% purity) was also obtained from Sigma-Aldrich, while demethoxycurcumin (dmc, >95% purity) and bisdemethoxycurcumin (bdmc, >95% purity) were purchased from Biopurify Chemicals Ltd. (Chengdu, Sichuan, China). LC–MS grade water, methanol and acetonitrile solvents, formic acid (98–100%) and dimethyl sulfoxide (DMSO) were also provided by Sigma-Aldrich.

Stock standard solutions of all targeted bioactive compounds (1000 mg/L) were prepared in methanol and were kept in amber glass vials. Working intermediate solutions were prepared by appropriate dilution of the stock standard solutions with water. All the solutions were stored in the refrigerator at 4 °C for not more than 1 month.

### 3.2. Instrumentation

An Accella liquid chromatography instrument (with a quaternary pump and an autosampler) from Thermo Fisher Scientific (San Jose, CA, USA) coupled to a Q-Exactive Orbitrap HRMS system (Thermo Fisher Scientific), equipped with a heated electrospray ionization source (HESI-II) was employed for the analysis of turmeric and curry samples. For that purpose, a reversed-phase porous-shell Ascentis^®^ Express C18 (150 mm × 2.1 mm I.D., 2.7 µm partially porous particle size) column provided by Supelco (Bellefonte, PA, USA) was used under gradient elution conditions with water (solvent A) and acetonitrile (solvent B), both with 0.1% formic acid, as mobile phase components at a flow rate of 0.3 mL/min. The column was maintained at room temperature. The gradient elution program was as follows: From 0 to 1 min, isocratic elution at 10% solvent B; from 1 to 20 min, linear gradient elution up to 95% solvent B; from 20 to 23 min, isocratic elution at 95% solvent B; then back to initial conditions at 95% solvent B from 23 to 24 min; and finally, an isocratic elution step from 24 to 30 min at 10% solvent B for column conditioning and re-equilibration for the next analysis. The injection volume was 10 µL in full loop mode.

HESI-II ionization source operated in negative ionization mode. HESI-II sheath, ion-sweep, and auxiliary gases were nitrogen, with a purity higher than 99.98%, at flow rates of 60, 0, and 10 arbitrary units (a.u.), respectively. Other H-ESI ionization parameters were as follows: capillary voltage, −2.5 kV; heater temperature, 350 °C; capillary temperature, 320 °C; and S-Lens RF voltage, 50 V. HRMS acquisition was performed in full scan mode from 100 to 1500 *m/z* at a 70,000 full width at half maximum (FWHM, at *m/z* 200) resolution. For full scan mode, the automatic gain control (AGC) target (the number of ions to fill the Orbitrap C-trap) was established at 2.5 × 10^5^, and the maximum injection time (IT) at 200 ms. A data-dependent scan mode consisting of a product ion scan acquisition was also employed. This acquisition mode was activated when a *m/z* full scan signal higher than 1.0 × 10^5^ was detected. Then, the data-dependent product ion scan mode was obtained by employing stepped normalized collision energies (NCE) of 17.5, 35 and 52.5 eV, by fixing at 50 Da the first *m/z* value of the registered product ion scan range, and by establishing a quadrupole isolation window of 0.5 *m/z*. For data-dependent product ion scan acquisition, a mass resolution of 17,500 FWHM at *m/z* 200, with AGC and IT values of 2.0 × 10^5^ and 200 ms, respectively, were used.

The Q-Exactive Orbitrap HRMS analyzer was tuned and calibrated every 3 days by using a calibration solution supplied by Thermo Fisher Scientific. Control of the LC-ESI-HRMS system and data processing was carried out by using Xcalibur^TM^ 2.2 software (Thermo Fisher Scientific).

### 3.3. Samples and Sample Treatment

A total of 30 commercial samples (21 turmeric and 9 curry samples) were obtained from local markets in Barcelona (Spain). Sample brands and characteristics are summarized in Table 2. All samples were analyzed in triplicate, thus giving a total of 90 sample extracts.

Extraction of bioactive substances from samples was carried out by using a previously described procedure with modifications [40]. For that purpose, 10 mg of turmeric or curry sample was accurately weighed and extracted with 5 mL of DMSO at room temperature by ultrasound-assisted extraction in a Branson 5510 bath (Branson Ultrasonics, Danbury, CT, USA) during 15 min. Then, samples were centrifuged for 15 min at 3500 × g (Rotanta 460 RS centrifuge, Hettich, Tuttlingen, Germany), and 2 mL of the supernatant solutions were filtered through 0.45 µm nylon membrane syringe filters (Whatman, Clifton, NJ, USA) and were kept in amber injection glass vials.

Regarding to the stability of the extracts, it is well known that curcuminoids and polyphenols are powerful antioxidant molecules so they are sensitive to the presence of oxidants, temperature, light exposure, etc. thus leading to different oxidized molecules, among which quinone species are often produced [56]. In order to minimize degradation, DMSO extracts were stored in the freezer at −18 °C until the LC-HRMS analysis. Under these circumstances, solutions were stable for, at least, 1 week without appreciating significant decreases in curcuminoid and polyphenol concentrations.

To evaluate the repeatability of the proposed methodology and the robustness of the chemometric results, a quality control (QC) solution was prepared by mixing 50 µL of each turmeric and curry sample extract.

All turmeric and curry samples were analyzed randomly with the proposed LC-HRMS method to prevent the variations that may be produced by the sample sequence duration. Besides, after every 10 analyzed turmeric and curry samples, a QC and an instrumental chromatographic blank (acetonitrile) were run to ensure the good performance of the proposed methodology.

### 3.4. Data Analysis

Targeted LC-ESI-HRMS polyphenolic and curcuminoid profiles were obtained by processing the LC-HRMS raw chromatographic data with TraceFinder^TM^ version 3.3 software from Thermo Fisher Scientific. For that purpose, two home-made accurate mass database lists were used: (i) list with 53 previously characterized polyphenol and phenolic compounds with HRMS and MS/HRMS data [53], and (ii) list with accurate HRMS *m/z* values of 24 curcuminoid (Table S1 in the Supplementary Materials), including curcumin, dmc and bdmc, as well as other curcumin-related derivatives (data obtained from commercially available standards and from literature). It should be mentioned that for most of the compounds, both keto and enol tautomers can be present and detected.

In all cases, confirmation criteria to assess the presence of the targeted bioactive substances in the turmeric and curry samples relied on accurate mass errors (values below 5 ppm) and isotopic pattern matches (scores higher than 85%). Besides, additional confirmation criterion based on the chromatographic retention time was also employed for the 53 polyphenolic compounds and for curcumin, dmc and bdmc.

Eigenvector Research Stand Alone Chemometric Software (SOLO) was employed for PCA and PLS-DA chemometric calculations [57]. The theoretical background of the chemometric procedures employed in this work are described elsewhere [58]. Plots of scores and loadings from PCA open up great opportunities to display chemical information in a quite simple and condensed way as principal components (PCs) are calculated to retain the maximum amount of chemical information from the data. More specific tasks, such as the classification of samples into predefined categories are also available from PLS-DA.

X-data matrices to be processed by PCA and PLS-DA were built from LC-HRMS polyphenolic and curcuminoid profiles as chemical descriptors, giving place to a data matrix with a dimension of 101 (sample extracts + QCs) × 135 (peak areas of the *m/z* targeted values at the different retention times detected). Normalization pretreatment regarding the overall analyte response was applied to provide similar weights to all the samples. The Y-data matrix used in the PLS-DA models was defined by the membership to the corresponding class of each sample. Information regarding correlations and dependences of the analyzed samples with the targeted compounds was visualized in the scatter plots of scores and loadings of principal components (PCs) or latent variables (LVs) from PCA and PLS-DA, respectively. The most appropriate number of LVs for each PLS-DA model was established from the first significant minimum of the error function by Venetian blind cross-validation (CV). Besides, the PLS-DA models were validated to prove their applicability to sample classification. For that purpose, 70% of the samples of each group were randomly selected as the calibration set, and the remaining 30% of the samples were employed as “unknown” samples in the prediction set.

## 4. Conclusions

In this work, targeted LC-HRMS polyphenolic and curcuminoid profiles, obtained by C18 reversed-phase chromatography after a simple liquid–solid sample extraction, have demonstrated to be excellent sample chemical descriptors to address the characterization and classification of turmeric and curry samples. Despite the complexity and cost of LC-HRMS, this technique is increasingly used in food analysis because of its excellent performance in the research of specific descriptors for characterization and authentication purposes.

Due to the huge amount of data generated from the analysis of the set of samples, the use of chemometric methods is almost essential to extract relevant chemical information. In this regard, the exploratory analysis performed by PCA and PLS-DA showed great discrimination capabilities among turmeric versus curry samples. In addition, 100% classification rates in both calibration and prediction steps were achieved by PLS-DA.

Among the targeted bioactive substances, curcuminoids and some phenolic acids such as *trans*-cinnamic, ferulic and sinapic acids, were essential for the discrimination of turmeric samples, while polyphenolic compounds, in general, were important to differentiate curry samples. However, it should be remarked that, despite the fact that curcumin and related derivatives are found at higher concentrations in turmeric samples in comparison to curry samples, both curcuminoid and polyphenolic profiles were necessary for sample exploration and classification.

Besides, and despite the low number of turmeric samples analyzed, certain discrimination among turmeric *Curcuma longa* vs. turmeric *Curcuma zedoaria* species, as well as between different *Curcuma longa* (Alleppey, Madras and Erode) varieties was also achieved when using targeted LC-HRMS polyphenolic and curcuminoid profiles as sample chemical descriptors.

The results obtained in this work demonstrate that the proposed LC-HRMS polyphenolic and curcuminoid profiling method is feasible for the characterization, classification and authentication of turmeric and curry samples. However, it should be commented that the authenticity and composition of the analyzed samples are based on the manufacturer declaration only.

## Figures and Tables

**Table 1:** TraceFinder^TM^ bioactive compound profiling report obtained for a selected turmeric (Biospirit brand) sample.

Target Name	+/−	Area	Formula	Expected *m/z*	Measured *m/z*	Error (ppm)	Isotopic Pattern Score (%)
*Polyphenolic accurate mass database*							
D-(-)-Quinic acid	−	2.37 × 10^6^	C7H12O6	191.0561	191.0565	2.19	100
Syringic acid	−	1.92 × 10^5^	C7H12O6	197.0455	197.0460	2.80	100
2,5-Dihydroxybenzoic acid	−	7.57 × 10^5^	C9H10O5	153.0193	153.0193	0.21	100
Caffeic acid	−	4.34 × 10^5^	C9H10O5	179.0350	179.0353	1.90	100
Homovanillic acid	−	6.09 × 10^5^	C7H6O4	181.0506	181.0510	2.27	100
4-Hydroxybenzoic acid	−	3.82 × 10^6^	C9H10O4	137.0244	137.0244	0.55	100
Homogentisic acid	−	2.10 × 10^6^	C7H6O3	167.0349	167.0351	1.06	100
Ellagic acid	−	3.26 × 10^5^	C8H8O4	300.9990	300.9996	2.00	93
*p*-coumaric acid	−	4.32 × 10^6^	C8H8O4	163.0401	163.0401	0.37	100
Ferulic acid	−	1.05 × 10^6^	C14H6O8	193.0506	193.0510	2.28	100
Vanillin	−	6.59 × 10^5^	C9H8O3	151.0401	151.0402	1.11	100
*trans*-Cinnamic acid	−	2.92 × 10^5^	C10H10O4	147.0452	147.0454	1.53	100
Rosmanol	−	1.05 × 10^6^	C8H8O3	345.1707	345.1711	1.28	100
Quercetin	−	1.56 × 10^5^	C20H26O5	301.0354	301.0357	1.22	91
Homoplantaginin	−	1.78 × 10^7^	C15H10O7	461.1089	461.1071	−3.74	83
Umbelliferon	−	1.11 × 10^5^	C15H10O7	161.0244	161.0242	−1.24	100
Carnosol	−	2.36 × 10^5^	C22H22O11	329.1758	329.1763	1.52	78
*Curcuminoid accurate mass database*							
1	−	9.80 × 10^6^	C19H18O5	325.1081	325.1085	1.33	100
N1	−	1.36 × 10^6^	C19H16O6	339.0874	339.0877	0.97	100
8	−	3.61 × 10^7^	C19H16O3	291.1026	291.1030	1.33	100
6	−	6.30 × 10^5^	C21H22O7	385.1292	385.1296	1.01	85
N11	−	1.43 × 10^8^	C20H18O4	321.1132	321.1135	1.01	100
5	−	2.62 × 10^7^	C19H16O5	323.0925	323.0928	0.94	100
N7	−	2.51 × 10^7^	C21H20O5	351.1238	351.1240	0.72	100
10	−	1.78 × 10^7^	C20H18O6	353.1030	353.1032	0.43	100
9	−	6.53 × 10^7^	C19H18O4	309.1132	309.1135	1.15	100
N2	−	1.78 × 10^7^	C20H20O5	339.1238	339.1241	0.93	100
bdmc	−	1.40 × 10^9^	C19H16O4	307.0975	307.0979	1.34	100
N3	−	3.29 × 10^7^	C21H22O6	369.1343	369.1346	0.77	100
dmc	−	1.44 × 10^9^	C20H18O5	337.1081	337.1087	1.82	100
curcumin	−	1.65 × 10^9^	C21H20O6	367.1187	367.1192	1.34	100

**Table 2:** Number of turmeric and curry samples analyzed and their characteristics.

Sample	Commercial Brand	Number of Samples *	Number of Extracts	Compositional Characteristics
Turmeric	Hacendado	5	15	*Curcuma longa* (Erode)
	MG	1	3	*Curcuma longa* (Alleppey)
	Burriac	1	3	*Curcuma longa*
	Carmencita	1	3	*Curcuma longa* (Erode)
	Ducros	1	3	*Curcuma longa*
	Artemis Bio	1	3	*Curcuma longa*
	Natco	1	3	*Curcuma longa*
	Pelotari	1	3	Unknown
	Dani	2	6	*Curcuma zedoaria*
	Especies	1	3	*Curcuma longa* (Alleppey)
	Ocena	1	3	*Curcuma longa* (Madras)
	Tata Sampann	1	3	Unknown
	Herbalist	1	3	*Curcuma longa* (Madras)
	Street market	1	3	*Curcuma longa* (Madras)
	Biospirit	1	3	*Curcuma longa*
	NAAI	1	3	Unknown
Curry	Hacendado	2	6	Turmeric, white pepper, coriander, ginger, cardamom, clove, cinnamon, anise, mustard
	Carrefour	1	3	Turmeric, pepper, coriander, ginger, cumin, fenugreek, laurel, fennel, mustard
	Species Kania	1	3	Turmeric, pepper, coriander, cumin, fenugreek, parsley, chili, garlic, fennel
	Condis	1	3	Turmeric, pepper, coriander, fennel, cumin, cayenne, garlic, anise
	Burriac	1	3	Turmeric, white pepper, coriander, ginger, cardamom, clove, cinnamon, anise, mace
	Eroski	1	3	Turmeric, coriander, cardamom, ginger, fenugreek, anise, garlic, clove, mustard
	Ducros	1	3	Turmeric, pepper, coriander, cumin, ginger, laurel, anise, garlic, clove, cinnamon, mace
	Street market	1	3	Unknown (ca 30% turmeric)

* Number of containers collected from different locations.

## Supplementary Materials

The following are available online, Figure S1: PCA score plot of PC1 vs. PC2 showing the behavior of QCs and analyzed samples when targeted LC-HRMS bioactive compound profiles were used as chemical descriptors. A total of 4 PCs were used to build the model, Figure S2: Enlargement of loadings plots depicted in Figure 3 with full description names. (a) LV1 vs. LV2 and (b) LV1 vs. LV2 vs. LV3 when using corrected targeted LC-HRMS polyphenolic and curcuminoid profiles as sample chemical descriptors, Figure S3: PCA score plot of PC1 vs. PC2 when only corrected targeted LC-HRMS curcuminoid profiles were used as sample chemical descriptors. A total of 4 PCs were employed to build the model, Table S1: HRMS curcuminoid accurate mass database employed with TraceFinder^TM^ software.
