# Supplementary material for: Characterization, Classification and Authentication of Turmeric and Curry Samples by Targeted LC-HRMS Polyphenolic and Curcuminoid Profiling and Chemometrics
Source: Molecules. 2020 Jun 26;25(12):2942. doi: 10.3390/molecules25122942 (PMC7355898; doi:10.3390/molecules25122942)
Supplement: Supplementary file 1 [file molecules-25-02942-s001.pdf]

Supplementary Material

Characterization, Classification and Authentication of Turmeric and Curry Samples by Targeted LC-HRMS Polyphenolic and Curcuminoid Profiling and Chemometrics

Nerea Núñez[1,*], Oscar Vidal-Casanella[1], Sonia Sentellas[1], Javier Saurina[1,2], and Oscar Núñez [1,2,3,*]

[1] Department of Chemical Engineering and Analytical Chemistry, University of Barcelona, Martí i Franquès 1-11, E08028 Barcelona, Spain; oscarvidalcasanella@gmail.com (O. V.-C.); ssentellas@hotmail.com (S.S.); xavi.saurina@ub.edu (J.S.)

[2] Research Institute in Food Nutrition and Food Safety, University of Barcelona, Recinte Torribera, Av. Prat de la Riba 171, Edifici de Recerca (Gaudí), Santa Coloma de Gramenet, E08921 Barcelona, Spain

[3] Serra Húnter Fellow, Generalitat de Catalunya, Rambla de Catalunya 19-21, E08007 Barcelona, Spain

* Correspondence: nnunezte10@alumnes.ub.edu (N.N.), oscar.nunez@ub.edu (O.N.); Tel.: +34-93-403-3706

Table S1. HRMS curcuminoid accurate mass data base employed with TraceFinder™ software.

Compounds	Chemical formula	Tentative $[M-H]^-$ structure	$[M-H]^-$ *m/z* calculated value
curcumin	$C_{21}H_{20}O_6$	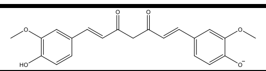	367.11869
Dmc	$C_{20}H_{18}O_5$	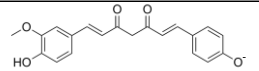	337.10812
bdmc	$C_{19}H_{16}O_4$	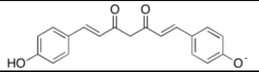	307.09756
5	$C_{19}H_{16}O_5$	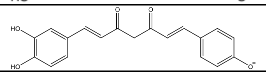	323.09247
10	$C_{20}H_{18}O_6$	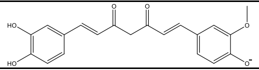	353.10304
N1	$C_{19}H_{16}O_6$	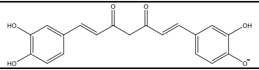	339.08739
1	$C_{19}H_{18}O_5$	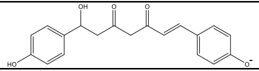	325.10812
2 and 3	$C_{20}H_{20}O_6$	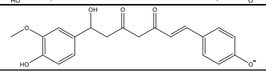	355.11869
6	$C_{21}H_{22}O_7$	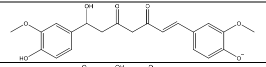	385.12925
4	$C_{19}H_{18}O_5$	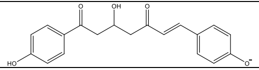	325.10812
9	$C_{19}H_{18}O_4$	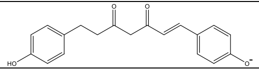	309.11321
N2	$C_{20}H_{20}O_5$	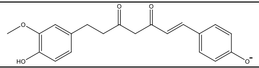	339.12377
N3	$C_{21}H_{22}O_6$	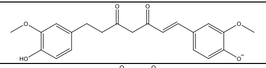	369.13434
N4	$C_{20}H_{20}O_6$	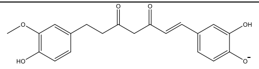	355.11869

N5	$C_{19}H_{18}O_6$	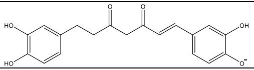	341.10304
N6	$C_{19}H_{18}O_5$	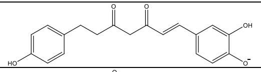	325.10812
8	$C_{19}H_{16}O_3$	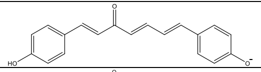	291.10264
N7	$C_{21}H_{20}O_5$	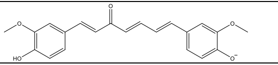	351.12377
N8	$C_{20}H_{18}O_5$	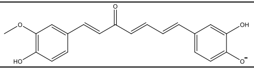	337.10812
N9	$C_{19}H_{16}O_5$	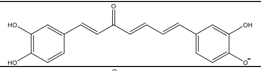	323.09247
N10	$C_{19}H_{16}O_4$	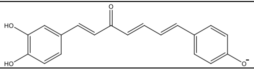	307.09756
N11	$C_{20}H_{18}O_4$	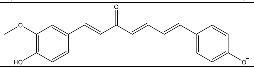	321.11321
7	$C_{18}H_{16}O_4$	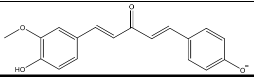	295.09756

For most of the compounds, both keto and enol tautormes can be present as shown below:

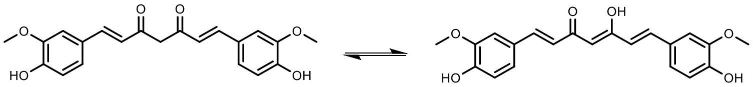

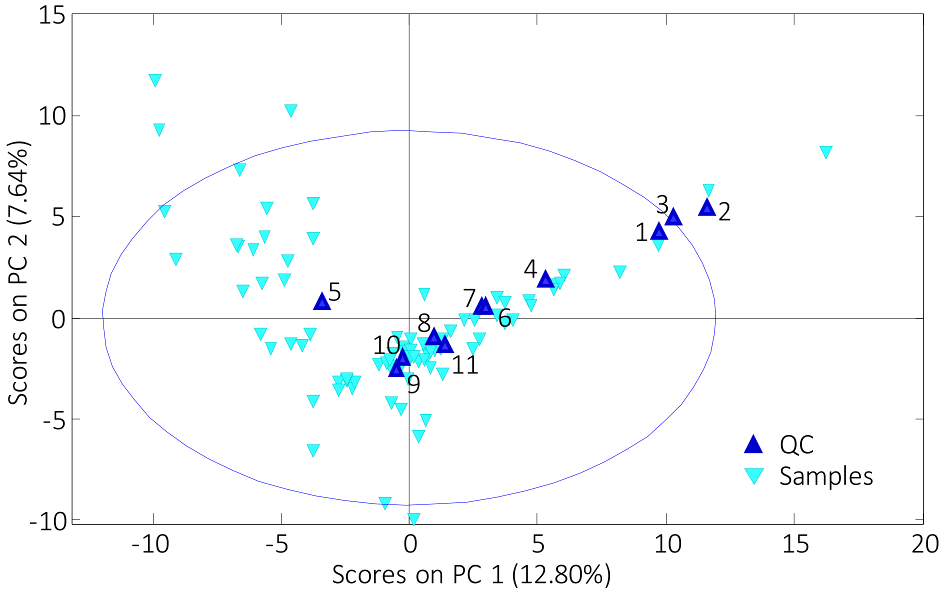

Figure S1. PCA score plot of PC1 vs. PC2 showing the behavior of QCs and analyzed samples when targeted LC-HRMS bioactive compound profiles were used as chemical descriptors. A total of 4 PCs were used to build the model.

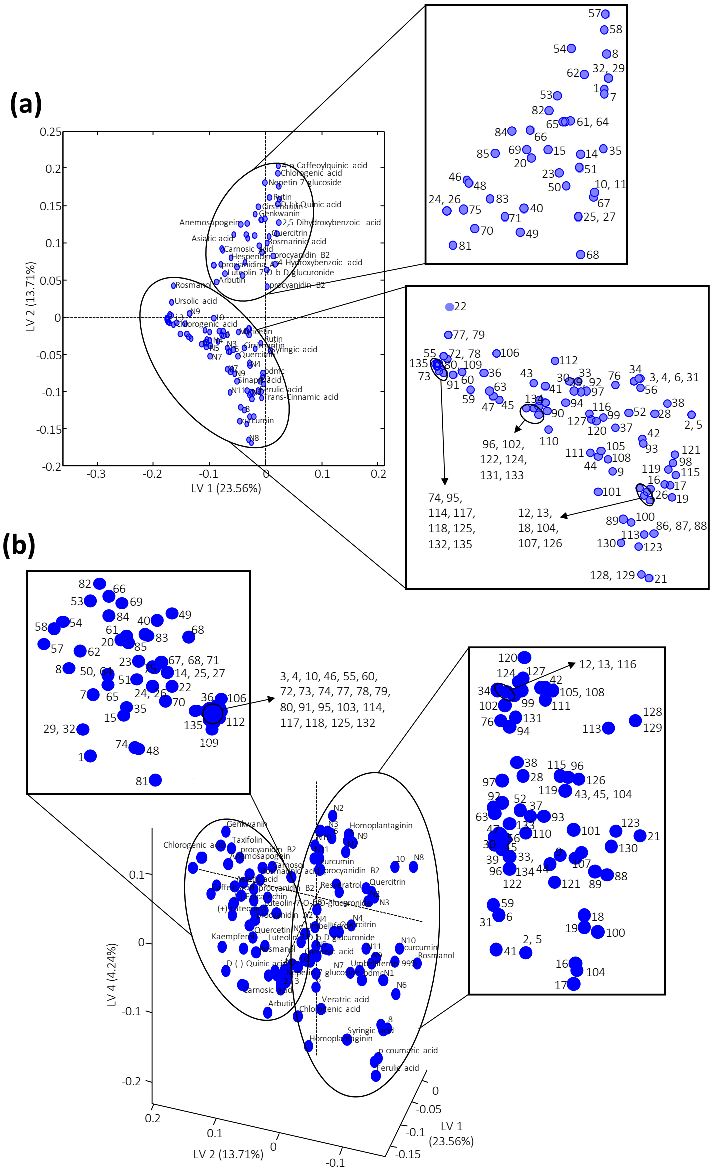

	Variable	t_R	68	procyanidin B2	6.27
1	D-(-)-Quinic acid	3.01	69	procyanidin B2	9.24
2	Ethyl gallate	3.81	70	Luteolin-7-O-b-D-glucuronide	5.73
3	Homovanillic acid	3.81	71	Luteolin-7-O-b-D-glucuronide	6.32
4	Syringaldehyde	3.81	72	Luteolin-7-O-b-D-glucuronide	10.55
5	Syringic acid	3.81	73	Luteolin-7-O-b-D-glucuronide	12.42
6	Veratric acid	3.81	74	Luteolin-7-O-b-D-glucuronide	13.22
7	2,5-Dihydroxybenzoic acid	3.82	75	procianidina A2	3.03
8	Caffeic acid	3.86	76	Myricetin	5.46
9	Sinapic acid	3.86	77	Betulinic acid	23.17
10	3,4-Dihydroxybenzaldehyde	4.32	78	Betulinic acid	25.46
11	4-Hydroxybenzoic acid	4.32	79	Ursolic acid	23.17
12	Homogentisic acid	4.36	80	Ursolic acid	25.46
13	Vanillic acid	4.36	81	Arbutin	3.02
14	Ellagic acid	4.49	82	Genkwanin	12.51
15	Polydatin	4.58	83	Hesperidin	3.96
16	*p*-coumaric acid	5.11	84	Anemosapogein	19.43
17	Ferulic acid	5.26	85	Asiatic acid	13.03
18	Vanillin	5.45	86	1	6.41
19	*trans*-Cinnamic acid	6.11	87	4	6.41
20	*trans*-Cinnamic acid	7.38	88	N6	6.41
21	Rosmanol	6.55	89	N1	6.86
22	Rosmanol	7.82	90	N1	7.23
23	Rosmanol	10.55	91	N4	7.92
24	Morin	4.01	92	N4	9.26
25	Morin	7.12	93	N4	10.89
26	Quercetin	4.01	94	N4	11.26
27	Quercetin	7.12	95	7	9.19
28	Resveratrol	7.47	96	6	8.85
29	Fisetin	7.07	97	6	9.91
30	Fisetin	8.77	98	6	10.81
31	Fisetin	9.33	99	6	11.16
32	Kaempferol	7.07	100	8	10.43
33	Kaempferol	8.77	101	N11	10.68
34	Kaempferol	9.33	102	N11	11.29
35	Quercitrin	4,00	103	5	9.44
36	Quercitrin	4.69	104	5	10.82
37	Quercitrin	10.95	105	5	11.42
38	Quercitrin	11.49	106	N9	9.44
39	Quercitrin	12.68	107	N9	10.82
40	Homoplantaginin	4.5	108	N9	11.38
41	Homoplantaginin	8.06	109	N7	10.90
42	Homoplantaginin	11.45	110	N7	11.44
43	Umbelliferon	11.67	111	N7	14.52
44	Umbelliferon	13.33	112	10	10.29
45	12-methoxycarnosic acid	17.13	113	10	11.14
46	12-methoxycarnosic acid	19.54	114	9	8.05
47	Carnosic acid	17.13	115	9	12.27
48	Carnosic acid	19.58	116	9	13.00
49	Carnosol	18.07	117	N2	8.03
50	Rosmarinic acid	3.83	118	N2	9.00
51	Rosmarinic acid	4.81	119	N2	12.54
52	Cirsimaritin	8.30	120	N2	13.14
53	Cirsimaritin	10.87	121	bdmc	12.80
54	Nepetin-7-glucoside	4.40	122	bdmc	13.47
55	Nepetin-7-glucoside	10.36	123	N10	12.70
56	Nepetin-7-glucoside	12.70	124	N10	13.47
57	4-o-Caffeoylquinic acid	3.97	125	N3	8.67
58	Chlorogenic acid	3.97	126	N3	12.83
59	Chlorogenic acid	9.93	127	N3	13.61
60	Chlorogenic acid	13.02	128	dmc	13.21
61	Gallic acid	3.79	129	N8	13.01
62	Rutin	3.65	130	curcumin	13.47
63	Rutin	12.61	131	curcumin	14.05
64	(-)-Epicatechin	3.97	132	N5	5.89
65	(+)-catechin	3.97	133	N5	6.66
66	Taxifolin	5.03	134	N5	7.49
67	Procyanidin B2	5.59	135	2 i 3	2.89

Figure S2. Enlargment of loadings plot depitect in Figure 3 with full name descriptios. (a) LV1 *vs.* LV2 and (b) LV1 *vs.* LV2 *vs.* LV3 when using corrected targeted LC-HRMS polyphenolic and curcuminoid profiles as sample chemical descriptors.

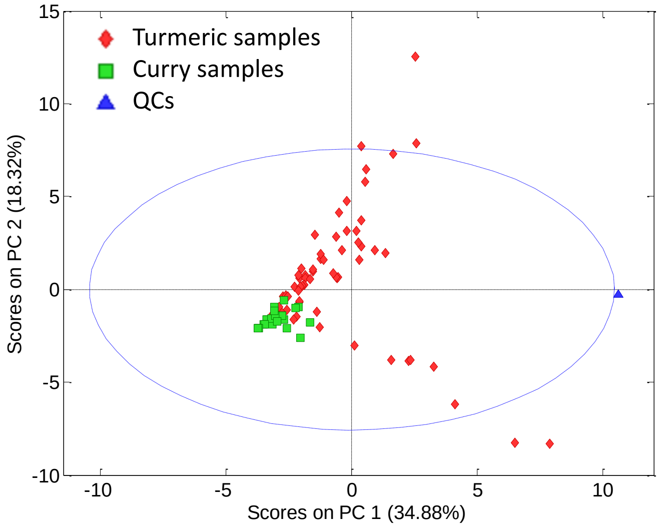

Figure S3. PCA score plot of PC1 vs. PC2 when only corrected targeted LC-HRMS curcuminoid profiles were used as sample chemical descriptors. A total of 4 PCs were employed to build the model.
